# More Bang for the Buck: Using miRNA-Treated Human iPSC-Derived Cardiomyocytes for Cardiac Repair∗

**DOI:** 10.1016/j.jacbts.2024.01.009

**Published:** 2024-05-27

**Authors:** Timothy J. Kamp

**Affiliations:** Department of Medicine, Stem Cell and Regenerative Medicine Center, University of Wisconsin, Madison, Wisconsin, USA

**Keywords:** cell cycle, human induced pluripotent stem cell, miR-590-3p, myocardial infarction

**Figure:**
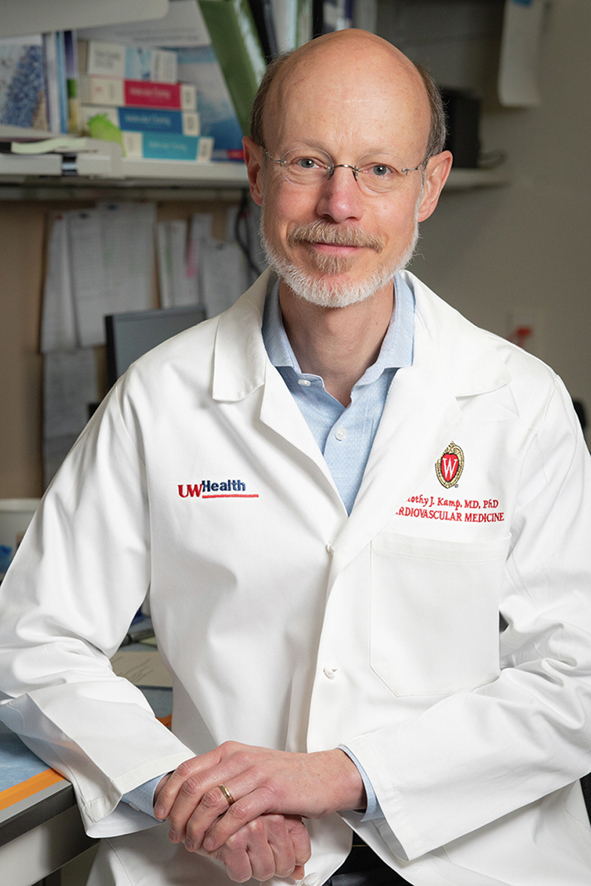

Although reperfusion remains the optimal therapy for acute myocardial infarction (MI), a substantial portion of patients are unable to achieve prompt reperfusion, leading to significant MIs. Following an MI, necrotic tissue is replaced by fibrous scar tissue rather than new functional muscle because the adult human heart lacks a regenerative capacity. Such loss of functional muscle can initiate the progressive spiral to heart failure and death despite optimal medical and device therapy. Although heart transplantation remains an option for end-stage heart failure, limited donor organs and lifelong immunosuppression remain major limitations. Thus, the quest to remuscularize the post-MI heart is being actively pursued using a variety of innovative approaches. Broadly, investigators are following 3 general strategies: 1) reactivate proliferation of endogenous cardiomyocytes; 2) reprogram native cardiac cells such as fibroblasts to functional cardiomyocytes; and 3) transplant human pluripotent stem cell-derived cardiomyocytes (hPSC-CMs) or cardiac progenitor cells. However, as the study by Zhang et al^1^ in this issue of *JACC: Basic to Translational Science* suggests, a combination of approaches may be the most effective.

Over the past decade, great progress has been made in developing approaches using hPSC-CMs to regenerate injured heart muscle in a variety of animal models. Despite a handful of phase 1 clinical trials testing the safety of various approaches to deliver hPSC-CMs to treat patients with heart failure, major roadblocks remain before the full potential of this approach can be realized—remuscularization of the damaged heart. Prominent among the challenges are: 1) overcoming immunological barriers for an allogeneic cell product; and 2) dosing and biomanufacturing limitations. The latter challenge relates to the fact that despite efforts at optimizing cell preparation and delivery approaches, intramyocardial delivery of hPSC-CMs leads to a relatively low survival rate for transplanted cells, <10% or lower. Thus, to realize significant remuscularization in large animal models, investigators have used large doses, generally in the range of 1 billion hPSC-CMs. Biomanufacturing approaches continue to improve, but the costs will potentially be prohibitive with current technology given that 1 billion hPSC-CMs must be generated per patient and more than 1 treatment may be needed. Thus, ways to enable smaller doses of hPSC-CMs to provide clinical benefit are of great practical importance to advancing this potential therapeutic avenue.

As in normal development, differentiation of hPSCs to CMs leads to cells that are initially proliferative, but as they mature, the cells exit the cell cycle and stop dividing. The hPSC-CMs that have been tested for cell therapy, although immature, have largely stopped proliferating. If instead of nonproliferating CMs, a population of proliferative CMs could be transplanted and the cells continue to divide, there could be more benefit per dose. A number of experimental approaches have been demonstrated to reactivate proliferation in quiescent CMs, including forced expression of certain microRNAs (miRs).^2^ MiRs are small RNAs of 18-22 nucleotides that are noncoding and typically act by binding the 3′-untranslated region of target messenger RNAs, leading to degradation and/or translational repression. Prior research showed that delivery of miR199a-3p or miR-590-3p in the post-MI adult mouse heart induced endogenous cardiomyocyte proliferation, reduced infarct size, and improved cardiac function.^2^ Can forced expression of proliferation-inducing miRs in hPSC-CMs maintain the hPSC-CMs in a proliferative state and improve dosing requirements?

Zhang et al^1^ tested AAV6-mediated delivery of miR-590-3p to hiPSC-CMs and demonstrated that forced expression of miR-590-3p did lead to increased proliferation of the hiPSC-CMs in culture. Furthermore, by characterizing the potential targets of miRNA-590-3p, they demonstrated that the target TSC22 domain family member 2 is down-regulated, which leads to up-regulation of pyruvate kinase M2 and downstream cell cycle genes. Next, the investigators tested delivery of hiPSC-CMs transduced with miR-590-3p to mouse hearts at the time of surgically induced MI. They found evidence using bioluminescent imaging of a luciferase reporter as well as histology that the miR-590-3p-expressing hiPSC-CMs formed larger grafts containing proliferating CMs at 28 days, which was accompanied by a significantly greater improvement in cardiac function relative to treatment with nontransduced hiPSC-CMs. The study then tested the same strategy delivering miR-590-3p hiPSC-CMs to pig hearts following surgically induced MI and showed remarkable improvements in cardiac function relative to sham control and improved relative to nontransduced hiPSC-CMs. This was accomplished in the porcine model with a dose of 50 million hiPSC-CMs, which is 10-20–fold lower than effective doses in prior large animal studies. Furthermore, the investigators did not find signals for toxicity, including a lack of induced ventricular tachycardia.

The finding that proliferating hiPSC-CMs are able to form larger grafts than nonproliferating hiPSC-CMs is consistent with recent studies that used an alternative approach to activate proliferation: overexpression of the key cell cycle gene, *CCND2*.^3^ Interestingly, the study by Zhao et al^3^ demonstrated that an effect of *CCND2* overexpression was to stimulate the release of exosomes containing miR302b-3p and miR373-3p, which increase the proliferation of CMs. But uncontrolled proliferation of hiPSC-CMs or native CMs can lead to adverse outcomes, as was highlighted by overexpression of miR-199a-3p in post-MI pig hearts resulting in sustained ventricular tachycardia and death despite significant cardiac regeneration.^4^ Thus, it will be essential to advance strategies that control the degree and timing of hiPSC-CM proliferation. Additionally, not all approaches to induce proliferation of hPSC-CMs will exert equal effects and safety. For example, miRs target large gene regulatory networks, and each “proliferation”-inducing miR may be associated with distinct risk profiles. Perhaps the fact that miR-590-3p has been demonstrated to inhibit proliferation of hepatocellular cancer cells and is being tested as an anticancer therapy suggests that it might help counter a concern from hPSC-related therapy: tumor formation.^5^ Additionally, therapy-induced ventricular tachycardia, whether primarily from the hPSC-CM graft or from continued proliferation of CMs, will be an important safety outcome that undoubtedly will differ among the distinct strategies. Although it is encouraging that Zhang et al^1^ did not observe ventricular tachycardia in the porcine post-MI model with 1 month of observation, longer and larger studies are needed to confirm this safety readout. Overall, exciting progress in advancing cardiac regeneration therapies to clinical application continues, but the biological complexities and practical realities require continued effort to fully realize the remarkable promise.

## Funding Support and Author Disclosures

The author has reported that he has no relationships relevant to contents of this paper to disclose.
